# Mumps Epidemiology and Mumps Virus Genotypes Circulating in Mainland China during 2013-2015

**DOI:** 10.1371/journal.pone.0169561

**Published:** 2017-01-13

**Authors:** Aili Cui, Zhen Zhu, Ying Hu, Xiuying Deng, Zhaodan Sun, Yan Zhang, Naiying Mao, Songtao Xu, Xueqiang Fang, Hui Gao, Yuan Si, Yake Lei, Huanying Zheng, Jilan He, Hongwei Wu, Wenbo Xu

**Affiliations:** 1 WHO WPRO Regional Reference Measles/Rubella Laboratory and Key Laboratory of Medical Virology Ministry of Health, National Institute for Viral Disease Control and Prevention, Beijing, People’s Republic of China; 2 Jiangsu Provincial Centers for Disease Control and Prevention, Nanjing, People’s Republic of China; 3 Heilongjiang Provincial Centers for Disease Control and Prevention, Ha’erbin, People’s Republic of China; 4 Shandong Provincial Centers for Disease Control and Prevention, Jinan, People’s Republic of China; 5 Shanxi Provincial Centers for Disease Control and Prevention, Taiyuan, People’s Republic of China; 6 Shannxi Provincial Centers for Disease Control and Prevention, Xi’an, People’s Republic of China; 7 Hubei Provincial Centers for Disease Control and Prevention, Wuhan, People’s Republic of China; 8 Guangdong Provincial Centers for Disease Control and Prevention, Guangzhou, People’s Republic of China; 9 Sichuan Provincial Centers for Disease Control and Prevention, Chengdu, People’s Republic of China; 10 Affiliated hospital of Beihua University, Jilin, People’s Republic of China; University of Hong Kong, HONG KONG

## Abstract

With the implementation of mumps virus (MuV) vaccination in the expanded program on immunization (EPI) in mainland China since 2008, the incidence of mumps has decreased, and the natural epidemic pattern of mumps has slightly changed during 2013–2015. The two epidemic peaks (April-July and November-December) became less obvious than those observed from 2004 to 2012. Children and adolescents younger than 15, particularly in the five-to-nine-year-old age group, remain the target group and should be the focus of high-quality immunization activities in mainland China. However, it was also found that the incidence and reported cases of mumps decreased in each age group during 2013–2015, particularly in the five-to-nine-year-old and ten-to-fourteen-year-old age groups. The proportion of mumps cases among adults in some provinces also increased. Unlike the changes in the epidemiological characteristics of mumps affected by vaccination, the data of MuV virology surveillance indicated that most of the MuV transmission chains have not yet been effectively interrupted, and MuV remains a natural epidemic pattern in mainland China. In the MuV virology surveillance, 194 MuV strains during 2013–2015 were isolated from 10 of 31 provinces in mainland China. Based on the phylogenetic analysis of the small hydrophobic (SH) gene, both genotype F (99.0%) and G (1.0%) were identified, and genotype F was still the predominant genotype continuously circulating in mainland China. Representative genotype F and G strains isolated in China from 1995 to 2012 were selected for further analysis. The results indicated that there were multiple transmission chains within genotype F, with no obvious geographical or time differences. The high genetic diversity of genotype F strains could be a result of the continuous transmission and evolution of the MuV in mainland China. Genotype G was also detected in four provinces in mainland China. Because of the limited epidemiological data, it was uncertain whether the genotype G MuV strains found in 2011 and 2013 were imported from other countries. Therefore, combined high-quality epidemiological and virological surveillance is necessary for mumps control; it can also be used to observe the changes in epidemiological characteristics and viral transmission of mumps over time after mumps-containing vaccine (MuCV) implementation and to provide a comprehensive epidemiological and genetic baseline for mumps elimination in mainland China.

## Introduction

Mumps is caused by the mumps virus (MuV) and is a type of acute respiratory infectious diseases that is prevalent worldwide. The inflammation and swelling of the parotid glands are the main clinical features of MuV infection, but the virus can also injure many internal organs and the central nervous system and cause the emergence of a variety of clinical manifestations, including pancreatitis, orchitis, deafness, sterile meningitis, encephalitis, and other complications.

MuV is a member of the genus *Rubulavirus* in the *Paramyxoviridae* family. The MuV genome is a non-segmented single-stranded negative strand RNA that contains 15,384 nucleotides. It encodes seven tandemly linked transcription units: the nucleo-(N), V/phosphor-/I (V/P/I), matrix (M), fusion (F), small hydrophobic (SH), hemagglutinin-neuraminidase (HN), and large (L) proteins [1,2]. Among them, the degree of variation of the SH gene is the largest in the entire genome and is therefore generally used as the basis for genotyping. MuV has been shown to have 12 genotypes (A-N, excluding E and M) circulating in the world [3], among which there is large diversity. In mainland China, genotype F has been the predominant MuV genotype, and it was also the native prevailing MuV genotype during 1995–2010 in mainland China [4].

Mumps is a vaccine-preventable disease. The vaccine is most often incorporated into national immunization programs in a combined measles-mumps-rubella (MMR) vaccine. In 2015, among 194 World Health Organization (WHO) member countries, 121 (62%) had incorporated MuV into their national immunization program, most of which used the MMR vaccine. In countries where large-scale immunization against mumps has been implemented, the incidence of the disease has dropped dramatically [5]. However, mumps outbreaks have recently reemerged in some areas and countries with high mumps immunization rates, which have caused wide concerns regarding its re-outbreak [6–10].

In mainland China, mumps vaccination began in the 1990s. However, the mumps vaccination rate was low at that time because of mumps vaccination of the self-supported and voluntary type in mainland China [11,12]. After 2008, a mumps-containing vaccine (MuCV) was formally introduced into the national immunization program, and the children received one dose of MuCV at 18–24 months of age. Both domestic and imported MuCVs were used in mainland China. The imported MuCV strain includes the Jeryl Lynn strain, which is one of the components of the trivalent MMR vaccine. The domestic MuCV strain was mainly composed of the S79 strain, which originated from the Jeryl Lynn strain and is used in the monovalent vaccine for mumps, the bivalent vaccine for measles and mumps (MM), and the trivalent MMR vaccine.

In this study, both epidemiological data on mumps and on variations in the MuV that circulated in mainland China from 2013 to 2015 were analyzed to understand the changes in the epidemiological characteristics and evolution pattern of MuV after implementation of the MuCV in 2008. The data also provide a scientific basis for the prevention and control of the disease.

## Results

### Epidemiology

**Fig 1:**
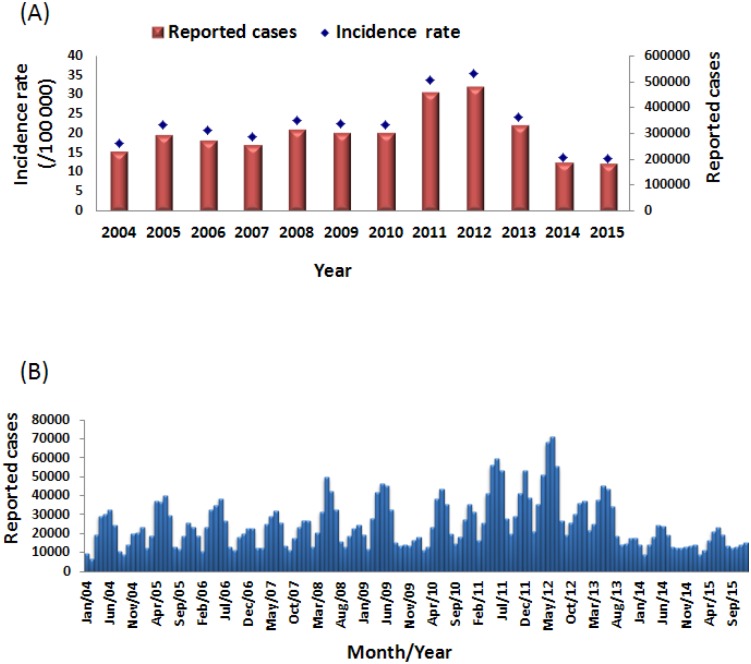
The mumps cases in mainland China by year (A) and by month (B) from 2004–2015.

In mainland China, the incidence of mumps in 2013, 2014 and 2015 was 24.21/100,000, 13.84/100,000, and 13.42/100,000, respectively. In these same years, there were 327,759, 187,500 and 182,833 reported mumps cases, respectively. The incidence rate of mumps gradually decreased from 2013 to 2015, and in 2015, the mumps incidence rate reached the lowest level during 2004–2015. In 2015, mumps incidence had declined by 62.29% compared to 2012 and the incidence had declined by 36.64% compared to the average incidence from 2004 to 2010 (21.18/100,000) (Fig 1A).

Mumps cases were reported throughout all 12 months of the year. There were obvious seasonal patterns, which are a well-known and described feature of mumps, and most of the mumps cases were concentrated between April and July, with small peaks also occurring in November and December in mainland China. With the decreased number of mumps cases in 2013–2015, the two epidemic peaks became less obvious than those observed from 2004 to 2012 (Fig 1B).

**Fig 2:**
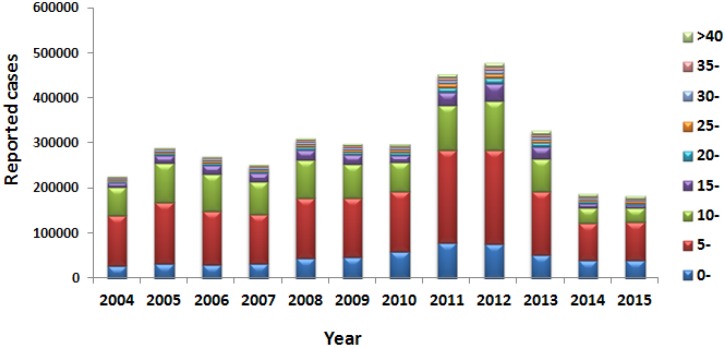
The age distribution of mumps cases in China from 2004–2015.

Most of the mumps cases occurred in primary and secondary schools. From 2013 to 2015, the majority of patients infected with mumps were children less than 15 years of age, particularly five-to-nine-year-old children. However, the incidence and reported cases of mumps decreased in each age group during 2013–2015, particularly in the five-to-nine-year-old and ten-to-fourteen-year-old age groups, compared to those from 2004 to 2012 (Fig 2).

### The distribution of MuV genotypes in China in 2013–2015

In total, 194 MuV strains were isolated from clinical specimens collected in 10 of 31 provinces in China from 2013 to 2015. The phylogenetic analysis indicated that 192 of the 194 MuV strains belonged to genotype F, which confirmed that genotype F was still the predominant mumps genotype in China from 2013 to 2015. In addition, after excluding some identical or very similar sequences of virus isolates, 39 of the 192 genotype F MuV strains isolated from 2013 to 2015 were selected based on their genetic variability (>1 nucleotide variation) as representative viruses for a phylogenetic analysis.

Genotype G was also found in China in 2013, and two genotype G MuV strains were isolated from sporadic mumps cases in Jiangsu province. In addition, nine genotype G MuV strains were also detected from outbreaks or sporadic cases in three provinces (Liaoning: 1 strain; Shannxi: 5 strains; Fujian: 3 strains) of China in 2011. The strains from Shannxi and Fujian were obtained from mumps outbreaks in primary schools, and the strains from Liaoning and Jiangsu were obtained from sporadic mumps cases. The epidemiological data available for all these cases were limited.

### Phylogenetic analysis and genetic diversity of MuV genotype F sequences

The genetic characteristics of the MuV strains isolated from 2013 to 2015 in China were analyzed and combined with the data of Chinese genotype F MuV strains during 1995–2012. Compared to other genotypes, a few specific nucleotides were found in certain positions on the SH gene in most of the MuV genotype F strains, including C^65^, C^105^, G^137^, T^200^, C^239^, and C^262^.

The nucleotide divergence of the 192 MuV strains during 2013–2015 ranged from 0–6.65% (0/316-21/316) based on the SH gene. Among the MuV genotype F strains detected in mainland China from 1995–2015, the highest diversity of the nucleotide sequences of the SH gene was up to 9.81% (31/316). The maximum variation in the SH gene sequences was noted between MuVi/Jiangsu.CHN/23.11/3-F and MuVi/Beijing.CHN/10.07-F. In the process of the variation and evolution of MuV, the diversity between the wild type circulating strains and the early strains isolated from 1995 to 1996 or the Jeryl Lynn strain (mumps vaccine strain) increased every year in China from 1995 to 2015 (Table 1). Most of the nucleotide substitutions present in the SH gene sequences were random mutations. However, some nucleotide substitutions, such as T→A^118^, T→C^138^, C→T/A^238^, C→T^249^, A→G^270^, G→A^284^, and C→T^291^, had increased in frequency with MuV evolution and remained in most of the isolated Chinese MuV genotype F strains, particularly from 2013 to 2015.

**Table 1:** The values of the P distance among genotype F mumps virus strains and the Jeryl Lynn strain (MuCV strain) in China.

Group	No.	P distance		
		Within groups	1995–1996	Jeryl Lynn strain
1995–1996	5	0.0333	/	0.1276
2001–2007	28	0.0251	0.0336	0.1280
2008–2010	32	0.0325	0.0381	0.1330
2011–2012	29	0.0356	0.0408	0.1374
2013–2015	39	0.0403	0.0426	0.1395

**Fig 3:**
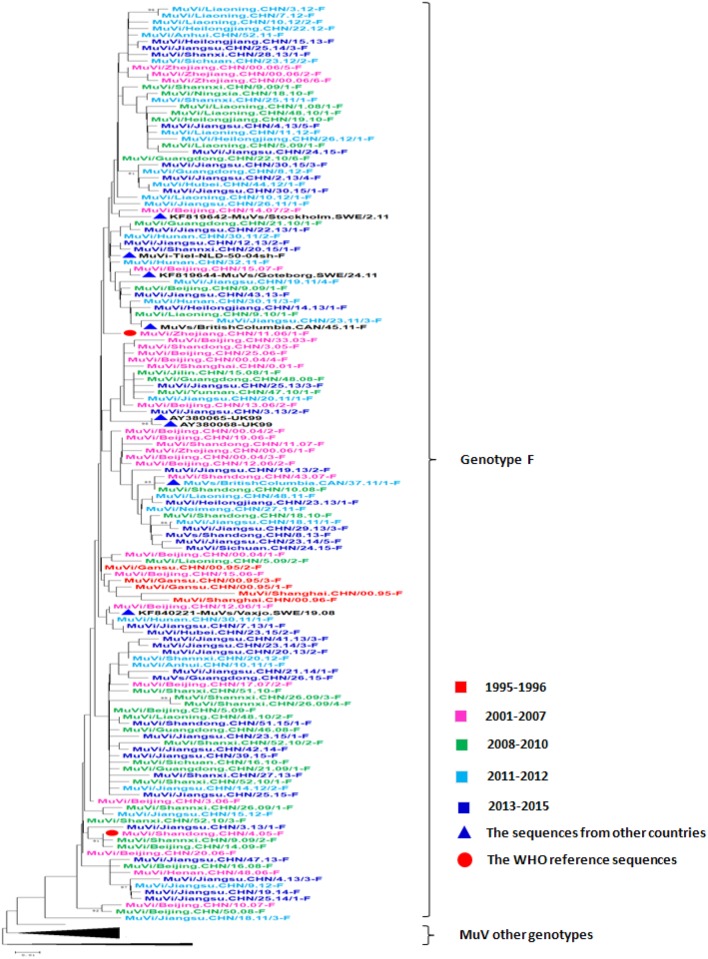
The phylogenetic analysis of the 39 representative Chinese genotype F MuV sequences from 2013–2015. The tree was drawn based on the SH sequence. The eight genotype F MuV sequences from other countries are indicated with solid blue triangles. The WHO reference virus sequences and the three mumps virus unclassified strains for genotyping obtained from GenBank are indicated with solid red dots.

The representative SH gene sequence dataset of MuV genotype F, including 39 isolates from 2013 to 2015, 92 isolates from 1995 to 2012, 8 isolates from other countries, and the 27 WHO references sequences, were used to analyze the genetic diversity of MuVs in mainland China over 20 years from 1995 to 2015 (Fig 3). The results of the phylogenetic analysis showed that multiple transmission chains were detected during this period, and the MuV strains from the transmission chains kept mutating simultaneously. The MuV strains isolated during 2013–2015 were interlaced with the strains from 1995 to 2012 in China, and no geographical restrictions or chronology correlations were found. For example, the identical sequence found in Shannxi, Liaoning, and Heilongjiang during the period of 2008–2010 was also detected in Jiangsu in 2011 and 2013. In addition, several genotype F MuV strains were also detected in other countries. These sequences of the SH gene were not clearly different from those from mainland China and crossed with Chinese sequences in the phylogenetic tree (Fig 3).

**Fig 4:**
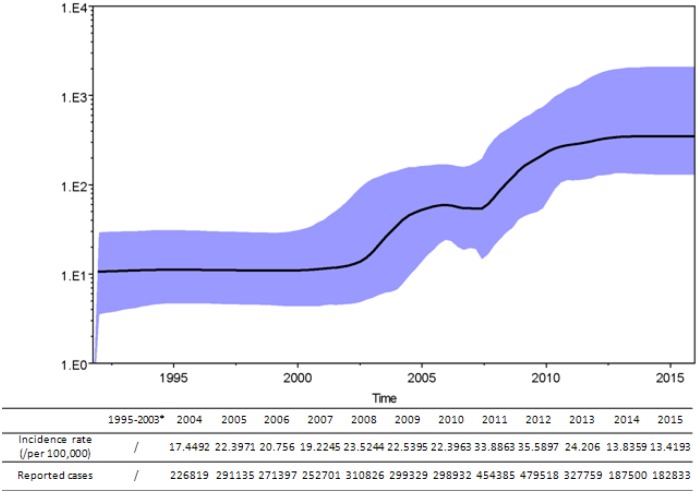
A Bayesian skyline plot analysis of the representative Chinese genotype F MuV sequences from 1995–2015. Ordinate: the number of effective infections at time; abscissa: time (in years). The thick solid line represents the median, and the blue area represents the 95% highest probability density (HPD) of the number of effective infections at the time estimates. The number of mumps cases according to the NNDRS is shown for each year below the skyline plot. *the data of the epidemiology surveillance of mumps during 1995–2003 were unavailable.

To independently estimate effective virus population sizes through time during 2013–2015, a BSP was used. The results showed minimal genetic diversity, likely due to an artifact caused by under-sampling from 1995 to 2003. Between 2003 and 2006, some genetic diversity was observed, which may be a consequence of the establishment of a surveillance program and better sampling throughout mainland China. Another burst in genetic diversity was observed between 2008 and 2012, likely because more locations were sampled and because mumps outbreaks occurred in China. During 2013–2015, the genetic diversity remained stable despite a significant decrease in the number of reported cases (Fig 4).

### Results of a phylogenetic analysis of MuV genotype G sequences

**Fig 5:**
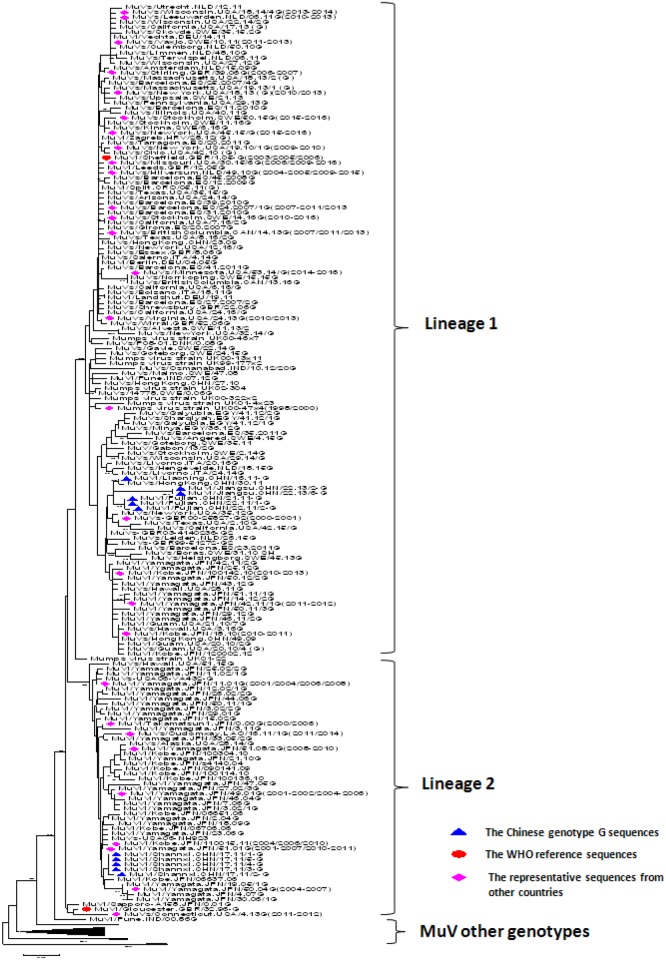
The phylogenetic analysis of the Chinese genotype G MuV sequences. The tree was drawn based on the SH sequence. The 11 Chinese genotype G mumps virus strains are indicated with solid blue triangles, and the WHO reference virus sequences and the three mumps virus unclassified strains for genotyping obtained from GenBank are indicated with solid red dots. The representative mumps virus strains collected from other countries deposited in GenBank are indicated with solid pink diamonds.

Nucleotide divergence of the SH gene between the 11 genotype G MuV strains detected in mainland China in 2011 and 2013 and the genotype G reference strains (MuVi/Gloucester.GBR/32.96-G and MuVi/Gloucester.GBR/32.96-G) was 2.22–5.38% (7/316-17/316). The diversity among these Chinese genotype G MuV strains was 0–6.96% (0/316-22/316). A phylogenetic tree was constructed with Chinese genotype G sequences and the sequences from other countries or regions downloaded from GenBank (Fig 5). Identical and similar sequences (single nucleotide variations) obtained from GenBank were deleted from the tree. In general, most of the genotype G sequences could be clearly divided into two lineages, which showed geographical differences. The sequences in lineage 1 were found in many countries of the world, including the United States, the Netherlands, Germany, Spain, the United Kingdom, Italy, Canada, Croatia, Denmark, India, Egypt, Gabon, Japan, and China, and most of them were found in Europe and North America from 1996 to 2016 [13–16]. The same sequences were also found in other countries. The sequences in lineage 2 were concentrated in Asian countries, including Japan, China, and Laos from 2000 to 2016 [17,18], but a few of them were also found in the United States in 2006 and 2014–2015. In addition, similar to genotype F, the MuV genotype G sequences from the different years and areas were intra-crossed within lineages 1 and 2 and did not show obvious differences in time or geography.

The 11 Chinese genotype G sequences belonged to different lineages in the phylogenetic tree. The sequences from Liaoning, Fujian, and Jiangsu were clustered together in lineage 1, which showed the highest homology with the sequences obtained from the UK in 2003 (EU606236) and Hong Kong in 2011 (KF031055). The MuV genotype G sequences from Shannxi belonged to lineage 2, which showed the highest homology with the sequences from Japan isolated in 2005 (AB699705). The nucleotide homology among all strains was 99%.

## Discussion

Although the MuCV was formally introduced into the national expanded program on immunization (EPI) in 2008, the mumps incidence in mainland China remained stable during 2008–2010 and was very similar to the incidence in the pre-vaccine period of 2004–2007. An epidemic occurred during 2011–2012, followed by a decreasing trend annually during 2013–2015 compared to the mumps incidence from 2004 to 2012. During 2013–2015, the age and seasonal distributions for mumps cases also changed slightly. Children younger than 15 years of age remained the most commonly infected population, but the reported mumps cases obviously decreased in the five-to-nine-year-old and ten-to-fourteen-year-old age groups. The number of mumps cases among adults also increased in some provinces [19]. In the epidemic season, the two epidemic peaks became less obvious in 2013–2015 than in the earlier period.

According to the MuCV strategy in mainland China, one dose of MuCV has been implemented among 18- to 24-month-old children since 2008. Before 2008, mumps vaccination was voluntarily, and recipients were vaccinated at their own expense. Because of the short time from the introduction of the MMR vaccine, the susceptible population of mumps infection was mainly concentrated in children of the five-to-nine-year-old age group, who had not yet received the mumps vaccination during 2004–2010. Thus, the mumps incidence did not change from 2004–2010 despite the introduction of the MMR vaccine in 2008. Moreover, the epidemic during 2011–2012 may also have resulted from the accumulation of many mumps-susceptible individuals and low mumps vaccination coverage because MuCV had only been introduced into the national immunization program for 3 to 4 years. The susceptible population of mumps infection was mainly concentrated in children in the five-to-nine-year-old age group, who had not yet received the mumps vaccination during 2011–2012. Therefore, the mumps vaccine did not affect the mumps epidemic in mainland China during 2008–2012. There were 2 possibilities for the changes of mumps epidemiological characteristics from 2013 to 2015. First, the susceptible populations decreased because of mumps outbreaks during 2011–2012; second, the mumps vaccination may have affected the epidemiology of MuV, which indicates that effective mumps vaccination is very beneficial for mumps control. Thus, continuing epidemic surveillances for mumps is necessary to understand whether the inclusion of MuCV will break the natural epidemic cycle of mumps in mainland China, and will help maintain the low incidence level.

Because China is a vast country and the regional economic development levels differ, each province can modify the supplementary immunization plan for mumps vaccination according to the present situation of regional characteristics and immunization programs in the region. Therefore, national MuCV coverage data are not available. The developed provinces, such as Beijing, Tianjin, Shanghai, and Zhejiang, can begin to implement 2-doses mumps vaccination series. The data showed that the annual incidences of these regions gradually declined each year with the second implemented dose of the MMR vaccine [19,20]. However, most children have only received 1 dose of MuCV in mainland China since 2008; thus, it is difficult to obtain a durable and strong immunity in the mumps-susceptible population. Moreover, the MMR vaccine is administered to 18- to 24-month-old children, and the high incidence age group (7–15 years old) cannot accept free vaccinations, affecting the vaccination rate.

WHO reported that MuCV coverage should reach 90% to prevent mumps outbreak[21]. Thus far, the mumps vaccination rate among children younger than 15 is low in most regions of mainland China, the two-dose vaccination rate is less than 10% in some areas [22–24], and a significant number of children have still not received immunity. In the MMR surveillance sites in Zhejiang in 2011, the data showed that approximately 70% of children aged 2–9 years were given at least one dose of MuCV, and no more than 40% of children aged 5–9 were immunized with two doses of MuCV [25]. A study in Chengdu city found that in the past 3 years, the current primary and middle school students in Chengdu reported a mumps vaccination rate of only 44.03%, far lower than the standards recommended by WHO and thus, prone to causing mumps outbreaks [26]. WHO recommend the use of two doses of the MMR vaccine in the process of eliminating measles, rubella and mumps and thus reducing the burden of disease. The use of live, attenuated MuCVs has nearly eliminated the disease from countries with high vaccine coverage rates of a two-dose regimen [5]. Thus, the timely inoculation of the second MuCV could provide another opportunity to the recipients, who might have had primary immune failure or be missing an inoculation. To improve the immunity level of the susceptible population and to reduce the incidence of mumps fundamentally, inoculation with two doses of MuCV is necessary in children under 15 years of age in mainland China.

Unlike changes in the epidemiological characteristic of mumps caused by vaccination, the data of MuV virology surveillance indicated that most of the MuV transmission chains have not yet been effectively interrupted, and MuV remains a natural epidemic pattern in mainland China, although the incidence of mumps has been reduced to a lower level after the implementation of MuV vaccination.

With the implementation of high-quality epidemiological surveillance for mumps, MuV virology surveillance also plays an important role in reducing the mumps epidemic because it can identify the source of the virus, track the transmission of the virus, and understand the genotype and variation of the representative strains in mainland China. Based on the data of MuV virology surveillance in mainland China, both genotype F and G were detected from 2013 to 2015. Genotype F was the predominant genotype over the previous 20-year period in mainland China, and multiple lineages of MuV were co-circulating. In addition, the MuV genotype F strains within the lineages showed no clustering tendencies in chronology or geography. For example, the same MuV with identical sequences was detected in several provinces in different years. Without sufficient immunization for the susceptible population, most of the transmission chains continue to spread the virus in mainland China. The diversity of Chinese genotype F mumps strains gradually increased during the twenty years between 1995 and 2015. Most of the substitutions in the SH gene were random mutations. However, some mutations were kept during the process of MuV evolution. The difference between the wild type genotype F MuV strains and the vaccine strain has also gradually increased, which might potentially affect vaccine effectiveness.

Genotype G MuVs were detected in 2011 and 2013 in mainland China. Unlike genotype F, genotype G has a global distribution and has been detected in many countries [27]. Based on the sequences data, two lineages of genotype G were found in the world, and these showed geographical differences. Lineage 1 was the global circulating virus during the period of 1996–2016 and was mainly found in Europe and North America during this time [13–16]. However, other countries/areas were also found to have the virus, including countries in Asia. Lineage 2 was concentrated in Asia, particularly in Japan [17,18]. The two genotype G MuV lineages were both found in China from outbreaks and sporadic cases. However, because of the limited epidemiological data available, we could not confirm whether these viruses were imported. The cases from Liaoning and Fujian had not received mumps vaccination, the vaccination history of the cases from Shannxi province were unknown, and the cases from Jiangsu province had received one dose of the MuCV but were still infected by MuV. The reinfection of the mumps cases in Jiangsu might indicated that one dose of MuCV could not effectively protect against mumps infection in the susceptible population with the waning of vaccine-induced immune responses; primary immune failure should also be considered [28].

Because mumps has a single serotype, the vaccine strains, which represent only a few of the known genotypes, can rationally protect against all genotypes of MuV wildtype strains infection. However, in vivo and in vitro studies suggest that cross-neutralization may be limited. Vaccinated persons develop sufficient neutralizing antibodies against wildtype MuV strains, although the neutralization capacity is lower against the wild type strain than it is against the vaccine strain [29,30].

Thus far, the S79 strain and the Jeryl Lynn strain were used as MuCV strains in mainland China; these strains belong to genotype A. The study data showed that the imported and domestic MuCV had no significant difference on vaccine effect. Moreover, the immune effect on the domestic vaccine showed that both the monovalent vaccine and the combined vaccine have good immunogenicity and a high seroconversion rate; additionally, the total protective effect of the vaccine was 70%, and the protective effect with 2 doses of MuCV was approximately 80% [22]. Mumps HI antibodies or the IgG antibody positive conversion rate was over 80% after 4 to 6 weeks of vaccination for children under 14 years old, who were negative for mumps antibodies before MuCV immunization [31]. A case-control study showed that the protective effects of 1 dose of MuCV were 92.6% for community sporadic cases and 68.2% for school outbreak cases in Guangzhou city [32]. Thus far, studies on cross-protection between genotype F MuV wildtype strains and the vaccine strain have rarely been reported. Therefore, the efficacy of neutralization of the predominant F MuV strains with the vaccine strain will be a focus of further study. In addition, in light of the prevalence of genotype F MuV stains in mainland China, the trial of a new mumps attenuated vaccine prepared from the F genotype strain has been reported, and the results demonstrated genotype-specific immunity induced by the MuCV immunization [33].

In conclusion, with the improved surveillance for MuV and high-quality immunization activities for mumps, multiple genotypes can be detected in the regions where the endemic transmission of MuV may be interrupted, which reflects the sources of mumps imported viruses, similar to what has been observed for the measles virus [34,35]. Combined high-quality epidemiological and virological surveillance is necessary for mumps control. This surveillance can demonstrate the changes of epidemiological characteristics and viral transmission of mumps over time after MuCV implementation and provide a comprehensive epidemiological and genetic baseline for mumps elimination in mainland China.

## Materials and Methods

### Epidemiological data

The numbers of mumps cases in this report were obtained directly from the National Notifiable Diseases Reporting System (NNDRS) of the China CDC.

#### Specimen collection and virus isolation

Throat swabs, oral fluid, and urine specimens were collected from clinically suspected mumps cases within seven days after the onset of symptoms during mumps outbreaks or sporadically during the period from 2013 to 2015. The isolation of MuV was performed using the Vero/hSLAM cell line [36], which was obtained from the US CDC (Global Specialized Laboratory for measles and rubella), and the cells were harvested when the visible cytopathic effect (CPE) covered at least 75–90% of the cell layer [37]. Virus isolation was conducted by provincial laboratories, and the isolates were transported to the National Measles Laboratory (NML) in Beijing for genotyping.

### RT-PCR amplification and sequence determination

Viral RNA was extracted from the virus suspension with CPE using the QIAamp mini viral RNA extraction kit (Qiagen, Valencia, CA, USA). RT-PCR was performed using a one-step RNA PCR kit (AMV) (TaKaRa, Japan) to amplify a 500-nt product containing the 316-nt WHO-recommended sequence window, as described previously [37]. The primers of SH5-1 (5’-AATATCAAGTAGTGTCGATGA-3’) and SH5-2 (5’-AGGTGCAAAGGTGGCATT GTC-3’) were used in the RT-PCR assay. PCR amplification for the SH gene was performed as follows: an initial incubation for 2 min at 94°C; 30 cycles of 45 sec at 95°C, 45 sec at 45°C, and 1 min at 72°C; and a final extension step for 10 min at 72°C. The amplification products were purified using a QIAquick Gel Extraction Kit (Qiagen), and the amplicons were bidirectionally sequenced using an ABI PRISM 3100 Genetic Analyzer (Applied Biosystems, Hitachi, Japan). To avoid the possibility of contamination of the RT-PCR, the separated RT-PCR stages were implemented using 4 different laboratories including an RNA exaction room, a reagent preparation room, an RT-PCR running room and a PCR treatment room. The negative and positive control were used for the RT-PCR assay.

### Phylogenetic analysis

The entire SH gene of the MuV strains, including the noncoding region (316 nt), was aligned to the WHO reference strains using Bioedit software (Version 7.0). Representative genotype F strains isolated in China from 1995–2012 [4], the WHO reference strains [3], and the MuCV strains (Jeryl Lynn strain) were downloaded from GenBank and selected for phylogenetic analysis in this study. The representative SH gene sequences of genotype G collected from other countries throughout the world were downloaded from GenBank. The phylogenetic trees for genotype F (Fig 3) and genotype G (Fig 5) were drawn on the basis of the SH gene using the neighbor-joining method in MEGA software (version 5.03) with bootstrapping (500 replicates). The substitution model was the Kimura 2-parameter for nucleotides.

In addition, a BSP (a retrospective model of population genetics) was used under both strict and relaxed clock conditions to estimate the demographic history with the 316-nt sequence window of the MuV sequences isolated from 1995–2015. This allowed for estimates of the effective population size over time with credibility intervals at every time point depending on errors due to the phylogeny reconstruction and the stochastic nature of the coalescent process [38].

### Nucleotide sequence accession numbers

The SH nucleotide sequences of 39 representative MuV genotype F strains and the 11 MuV genotype G strains isolated in this study were deposited into the GenBank database under accession numbers KX987600- KX987649. All of the strains were named according to the WHO nomenclature for the MuV.

### Ethics statement

In this study, the only human materials used were throat swabs, oral fluid and urine specimens collected from the clinically suspected mumps patients for the purpose of public health and disease control. This study was approved by the second session of the Ethics Review Committee of the National Institute for Viral Disease Control and Prevention in the China CDC and the methods were performed in accordance with the approved guidelines. Written informed consent for the use of the clinical specimens was obtained from all of the patients involved in this study.
